# Human and animal botulism surveillance in France from 2008 to 2019

**DOI:** 10.3389/fpubh.2022.1003917

**Published:** 2022-11-24

**Authors:** Sophie Le Bouquin, Camille Lucas, Rozenn Souillard, Caroline Le Maréchal, Karine Petit, Pauline Kooh, Nathalie Jourdan-Da Silva, François Meurens, Laurent Guillier, Christelle Mazuet

**Affiliations:** ^1^French Agency for Food, Environmental and Occupational Health & Safety (ANSES), National Reference Laboratory for Avian Botulism, Ploufragan-Plouzané-Niort Laboratory, Ploufragan, France; ^2^ANSES, Risk Assessment Department, Maisons-Alfort, France; ^3^Sante Publique France (French Public Health Agency), Direction des Maladies Infectieuses, Saint Maurice, France; ^4^French National Research Institute for Agriculture, Food and Environment (INRAE), Oniris, Unit of Biology, Epidemiology and Risk Analysis in Animal Health (BIOEPAR), Nantes, France; ^5^Department of Veterinary Microbiology and Immunology, Western College of Veterinary Medicine, University of Saskatchewan, Saskatoon, SK, Canada; ^6^Institut Pasteur, National Reference Center for Anaerobic Bacteria and Botulism, Université Paris Cité, Paris, France

**Keywords:** botulism, poultry, wild bird, One Health, surveillance, bovine

## Abstract

Botulism is a human and animal neurological disease caused by the action of bacterial neurotoxins (botulinum toxins) produced by bacteria from the genus *Clostridium*. This disease induces flaccid paralysis that can result in respiratory paralysis and heart failure. Due to its serious potential impact on public health, botulism is a closely monitored notifiable disease in France through a case-based passive surveillance system. In humans, this disease is rare, with an average of 10 outbreaks reported each year, mainly due to the consumption of contaminated foods. Type B and to a lesser extend type A are responsible for the majority of cases of foodborne botulism. Each year, an average of 30 outbreaks are recorded on poultry farms, about 20 cases in wild birds and about 10 outbreaks in cattle, involving a large number of animals. Mosaic forms C/D and D/C in birds and cattle, respectively, are the predominant types in animals in France. Types C and D have also been observed to a lesser extent in animals. With the exception of botulinum toxin E, which was exceptionally detected throughout the period in wild birds, the types of botulism found in animal outbreaks are different from those identified in human outbreaks over the last ten years in France and no human botulism outbreaks investigated have been linked to animal botulism. In line with the One Health concept, we present the first integrative approach to the routine surveillance of botulism in humans and animals in France.

## Introduction

Botulism is a neurological disease common to humans and animals, caused by the action of botulinum toxins (BoNT) produced by bacteria from the genus *Clostridium*. There are seven BoNTs described historically, identified from A to G. Human botulism is mainly associated with toxins A, B, E and F (1) and animal botulism with toxins C, D and the mosaic forms C/D and D/C (1, 2). BoNT are recombinant BoNT types. BoNT C/D is composed of the light chain of BoNT C and the heavy chain of BoNT D and BoNT D/C is composed of the light chain BoNT D and the heavy chain of BoNT C (3). Botulism occurs on all continents and is variable in incidence. In all species, the disease presents with flaccid paralysis, including respiratory and heart failures (1). Animal botulism affects many species, mainly birds and cattle in France (4), but also fur animals (i.e., minks or foxes) in northern European countries (5, 6) and horses in the United States (7). Based on the current knowledge available, intoxication is the main mode of contamination of cattle at the origin of clinical signs. It is therefore the ingestion of preformed toxins in food, water or any contaminated substance that is currently considered the cause of botulism. Avian botulism is the result of consumption of *Clostridium botulinum* spores. It is assumed that toxin production occurs *in vivo*. Ingested spores germinate, proliferate and produce toxin primarily in the cecum. Absorption of toxin formed in the digestive tract is responsible for the symptoms (8). In humans, it is a rare disease. Five types of botulism are typically described in humans, depending on the mode of contamination and exposure to the toxin: foodborne botulism, intestinal botulism, wound botulism, iatrogenic botulism and inhalational botulism (9, 10). Foodborne botulism and infant intestinal botulism are the two most common forms observed (11).

Animal botulism is considered an emerging problem in Europe (12). At the European level, botulism is monitored through the surveillance of zoonoses and zoonotic agents and the protection of workers (exposure to biological agents at work). In France, the regulatory framework requires mandatory official notification, both in humans and in animals, regardless of the species affected. Human botulism has been monitored by the French health authorities since the establishment of the National Reference Center for Anaerobic Bacteria and Botulism (NRC, *Institut Pasteur de Paris*) in 1978 and reporting the disease to *Santé Publique France* (SPF) has been compulsory since 1986. Any suspicion of human botulism requires notification to the regional health agency (ARS) and its biological confirmation by the NRC. In animals, botulism has been regulated since 2006, first in poultry and then in wild birds and cattle. Until then, it was classified as a first category health hazard for all susceptible species (13). With the promulgation of the Animal Health Law at European level in 2016 (14), the status of this disease has changed, because it does not appear as such in the list of diseases transmissible to animals or humans that must be subject to fixed prevention and control measures. A National Reference Laboratory for avian botulism was designated in France in 2011 (NRL, ANSES Ploufragan-Plouzané-Niort Laboratory).

Case reports of human and animal botulism are regularly published, but studies compiling surveillance data on botulism are scarce, particularly with respect to animal botulism.

Here, we present the results of human and animal botulism surveillance based on SPF data as well as NRC and NRL biological investigations. First, annual variability in the occurence of botulism is discussed, followed by a description of the outbreaks observed.

## Materials and methods

### Definitions

Before analyzing the surveillance data, it is important to note the differences in definition between the terms “case” and “outbreak” of botulism in human and animal health. In human health, a case of botulism refers to a single individual, whereas an outbreak of botulism refers to one or more individuals infected from a single source. In animal health, the terms case and outbreak refer to two different animal populations, regardless of the number of animals involved: the term case is only used for infections in wildlife, and the term outbreak is used for infections in domestic animals.

The incidence rate defined as the number of cases per 100,000 habitants was used in the following analysis for human botulism considering a French population of 65,9 millions over the 2008–2018 period according to Insee data (15).

This terminology will be used throughout the article.

### Data availability and study periods

Historically, the NRC diagnosed botulism in both humans and animals. In response to the sharp increase in the number of outbreaks reported on poultry farms in the late 2000s (16), an NRL for avian botulism was created at the ANSES Ploufragan Laboratory (Brittany, France) in 2011. Since then, some of the animal diagnoses have been carried out there, first on poultry and now also on wild birds. In 2017, the NRL also started to diagnose outbreaks in cattle. Here, this summary presents the results of human botulism surveillance based on epidemiological data from Santé Publique France (SPF, the French Public Health Agency) and the NRC's biological investigations, and those of animal botulism based on confirmed cases transmitted by the two reference laboratories, the NRC and the NRL. All reports of human botulism are recorded by the French health authority through SPF and human cases are confirmed by the NRC. These data concern metropolitan France and overseas. However, suspicions of animal botulism are not always confirmed or even tested, in particular those involving wild birds. Our analysis covers the period since 1987 with a focus on 2008–2018 for human botulism (17–19) and the period since 2005 with a focus on 2009–2019 for animal botulism. It was not possible to study exactly the same period in humans and animals. Nevertheless, the period considered for both covers a decade. A complementary analysis was carried out using NRL data to provide a more detailed description of the characteristics of the disease and its occurrence in animals since 2013.

### Diagnostic methods

Given that the symptoms are usually very typical, a presumptive diagnosis can be made on the basis of clinical findings alone, regardless of the species. However, several diseases are included in the differential diagnosis, and laboratory investigations are requested for the definitive diagnosis. In humans, the confirmatory diagnosis is based on the detection and identification of BoNT in serum and stool and/or the detection of the neurotoxigenic bacterium *C. botulinum* and some strains of *Clostridium baratii* and *butyricum* in stool or gastric contents. The bacterium and its toxin can also be tested for in suspect foods (20). The gold standard for the diagnosis of botulism is the mouse bioassay (21). Alternative methods such as Endopep-MS (22) have been developed, but are not currently used in France for the diagnosis of human botulism.

As in humans, clinical signs of animal botulism are evocative but not specific and are part of a differential diagnosis. Laboratory analyses are required to confirm the diagnosis established on clinical signs. There is no standard for the diagnosis of animal botulism and several laboratory methods are used. As in humans, the aim is to detect either the BoNT or BoNT-producing clostridia (23). Detection of BoNT-producing clostridia, often conducted using polymerase chain reaction (PCR) tools, could be questioned as this bacteria is ubiquitous. Based on the low prevalence of samples collected from asymptomatic animals and providing positive PCR results (24–26) compared to the high prevalence detected in animals with signs of paralysis (27), detection of BoNT-producing clostridia appears to be a valuable diagnostic strategy (23). Before 2010, diagnosis of animal botulism in France generally involved detecting BoNT in serum using the mouse bioassay (28), method that has been considered as the gold standard for laboratory confirmation of botulism for a long time. However, this bioassay does not discriminate between mosaic forms and non-mosaic forms. Today, the approach commonly used in France to confirm animal botulism is the detection of *C. botulinum* in biological samples such as feces, digestive contents as well as organs using PCR after an enrichment step in anaerobic broth (2, 29, 30). This choice has been made on the basis of the efficiency of this approach (user-friendly, time-saving, cost, ethical aspects) for detecting BoNT-producing clostridia in animals with clinical signs.

### Statistical methods

The variability of the number of human botulism cases was analyzed using the R incidence package (31). The log-linear regression model of the package was used. The fitted model is of the form log(*y*) = *r* × *t* + *b* where *y* is the incidence, *t* is the number of year since the first year of the analysis, and *b* is the intercept. The value of the parameter *r* characterizing the annual growth rate and its 95% confidence interval was determined using the fit() function of this package.

The results and graphs for animal botulism were produced in R (32), R-4.1.1 version using the ggplot2 package (33). The networkD3 package (34) was used for preparing Sankey diagrams.

### Food description

Foods involved in human botulism outbreaks were described with Foodex2 terminology (20). The foods at the origin of the outbreaks were described with term and facet as detailed as possible. FoodEx2 was also used to defined groups of food and the production method (see Supplementary material 1).

## Results

### Occurence of human and animal botulism cases and outbreaks in France

#### Human botulism

**Figure 1:**
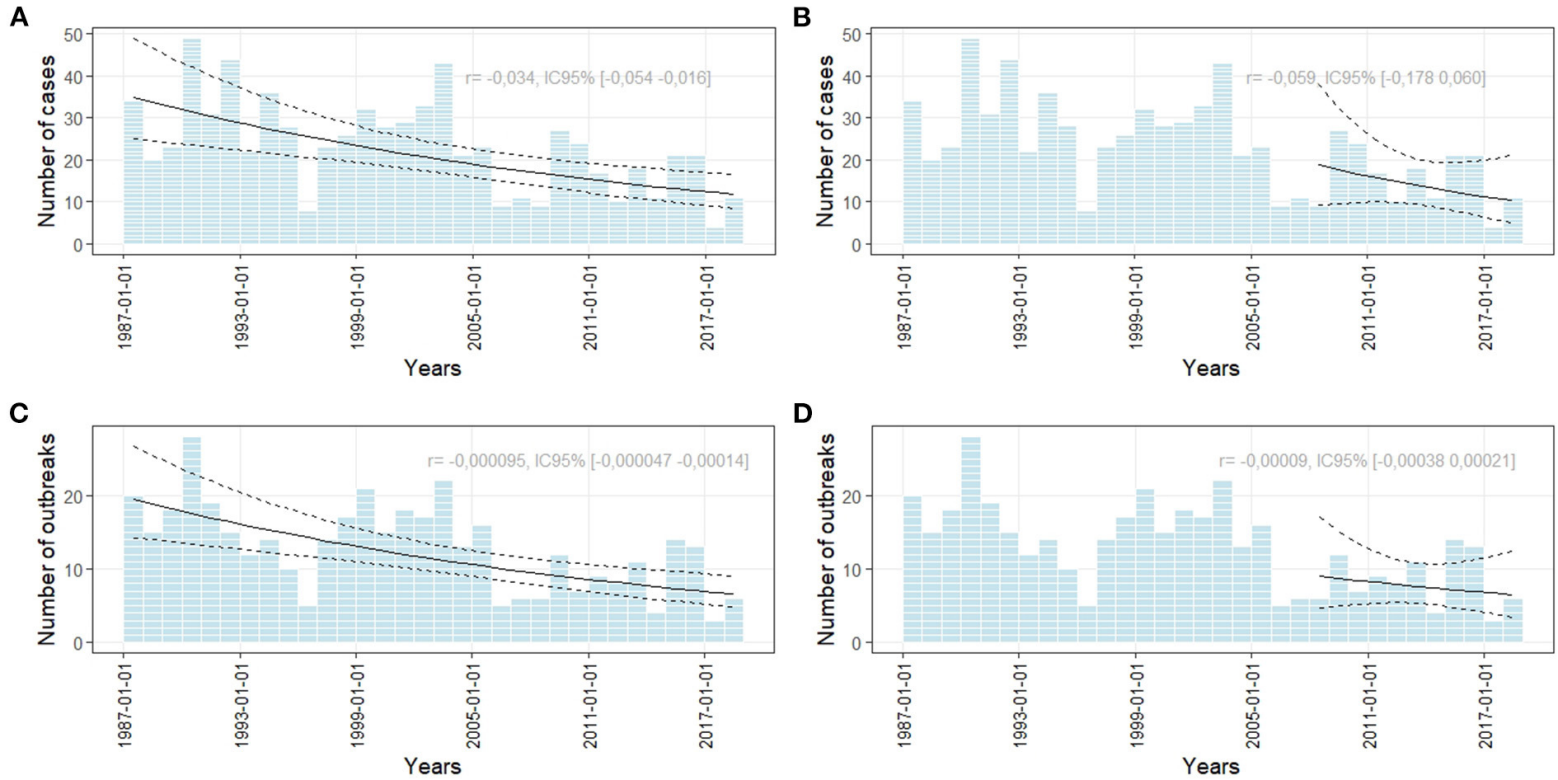
Number of foodborne human botulism outbreaks and cases based on NRC data. The curves represent a trend analysis over the period 1987–2018 **(A,C)** and over the period 2008–2018 [panel **(B,D)**]. *r* represents the growth rate of the log-linear model used for assessing the growth or decline of the number of cases or oubreaks.

Figure 1 shows the number of cases of human botulism observed since the establishment of an official surveillance system in France. The number of outbreaks appears to have decreased significantly during the 1987–2018 period (Figures 1A,C). The annual number of cases and outbreaks of foodborne human botulism in France has remained stable over the last 10 years (Figures 1B,D) with an incidence rate of 0.02 per 100,000 population. The annual number of outbreaks ranged from 3 to 13 outbreaks (average of 7.5 outbreaks) and for the number of cases per year from 4 to 25 (average of 14.5 cases). Of the 100 outbreaks of human botulism during the 2008–2018 period, 82 (89.8% of cases) were foodborne, 17 (9.6% of cases) were cases of infant intestinal botulism and 1 (0.6% of cases) were a wound botulism case observed in 2008 following an open leg fracture in a road traffic accident. No cases of infectious botulism in adults (intestinal colonization) were observed. The 82 foodborne botulism outbreaks represented a total of 159 cases. The maximum number of people involved in a single outbreak was six.

#### Animal botulism

**Figure 2:**
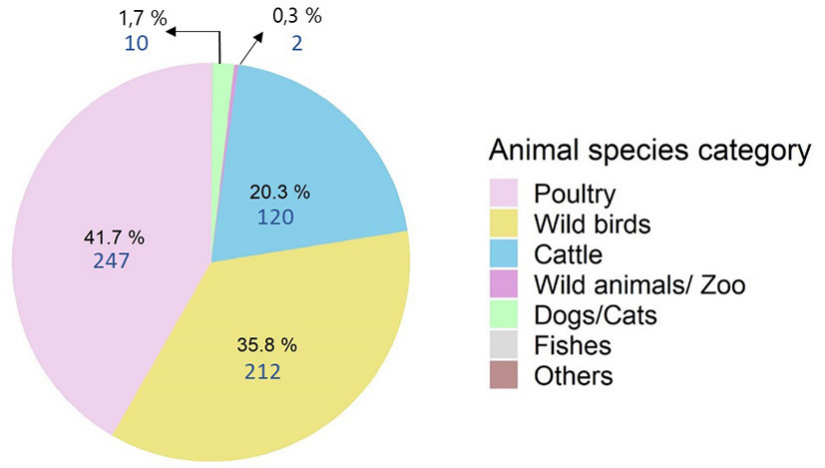
Distribution of animal botulism outbreaks from 2009 to 2019 by species (*n* = 592).

For the 2009–2019 period, 592 outbreaks of animal botulism were observed (Figure 2). Botulism was mainly detected in poultry (*n* = 247 or 41.7%), wild birds (*n* = 212, 35.8%) and cattle (*n* = 120, 20.3%). There were also a few outbreaks in dogs/cats between 2010 and 2015 (*n* = 10), fish in 2014 (*n* = 1) and wild/zoo animals in 2009 and 2011 (*n* = 2). Only the three major animal categories (poultry, wild birds and cattle) were analyzed in this study.

**Figure 3:**
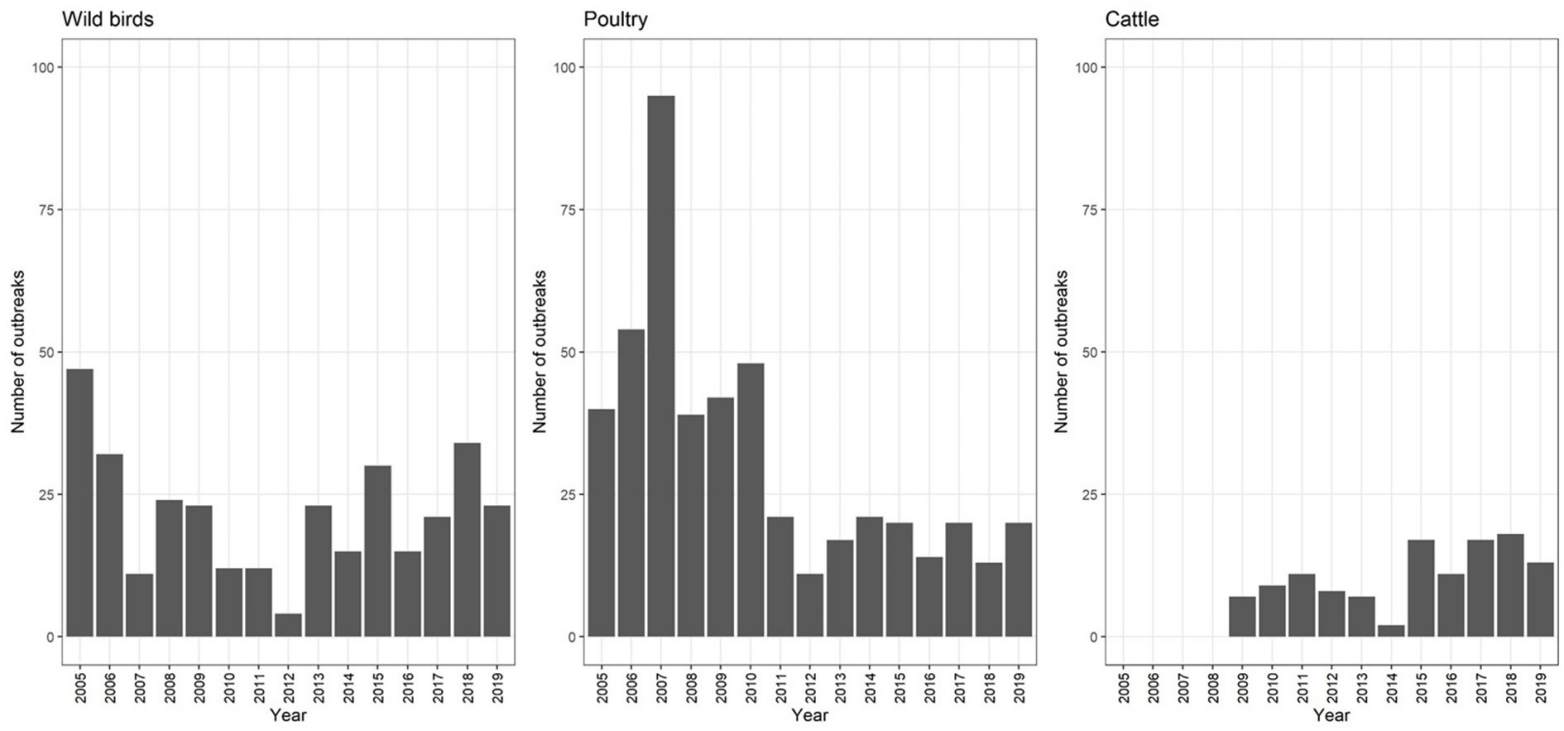
Evolution of the number of botulism cases in wild birds (2005–2019), outbreaks in poultry (2005–2019) and cattle (2009–2019) (*n* = 592).

The annual average number of outbreaks in poultry farms recorded between 2005 and 2011was 53.0 (SD = 21.3), with a sharp increase in 2007 when a peak of 95 outbreaks was observed (Figure 3). The origin of this peak has never been identified. Since 2011, this number has decreased to an average of 17.4 (SD = 3.8) outbreaks per year. Each year, an average of 21.7 (SD = 11.0) cases are recorded in wild birds and 10.9 (SD = 5.0) outbreaks in cattle. However, this number fluctuates from year to year.

### Description of botulism cases and outbreaks

#### In humans

**Figure 4:**
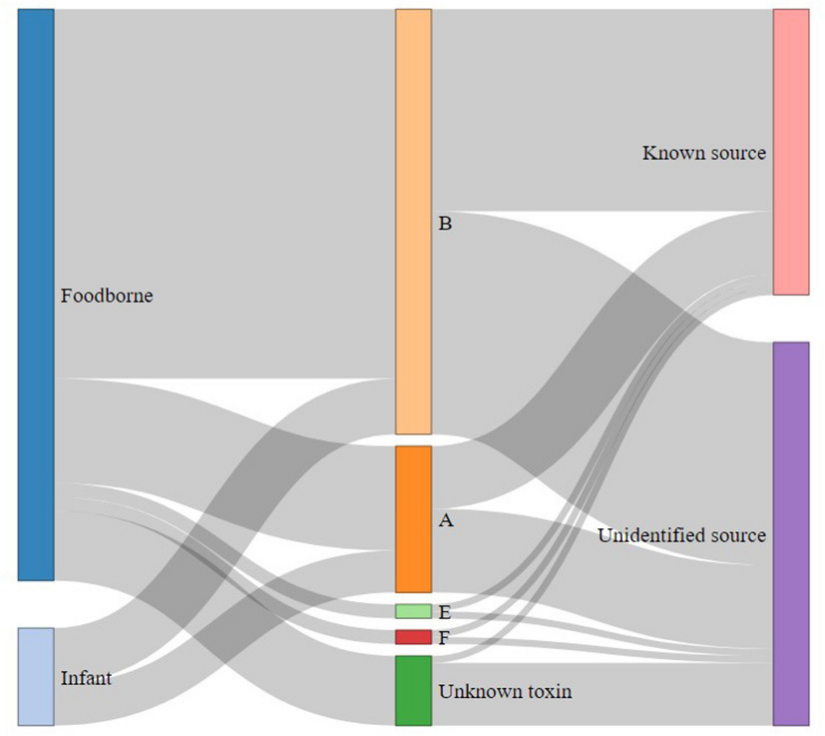
Distribution of human outbreaks (*n* = 82) and infant botulism outbreaks (*n* = 14) according to botulinum toxin type and case origin identification over the period 2008–2018 in France.

Over the period 2008–2018, type B was responsible for 53 (64%) outbreaks and 106 (67%) cases of foodborne botulism and type A for 15 (18%) outbreaks and 30 (19%) cases (Figure 4). Types E (two outbreaks) and F (two outbreaks) were responsible for four outbreaks involving four and five cases, respectively. Finally, for 10 outbreaks (14 cases) it was not possible to determine the BoNT type involved in the outbreaks or the cases (due to missing, insufficient or delayed biological samples, or unidentified or unavailable food).

**Figure 5:**
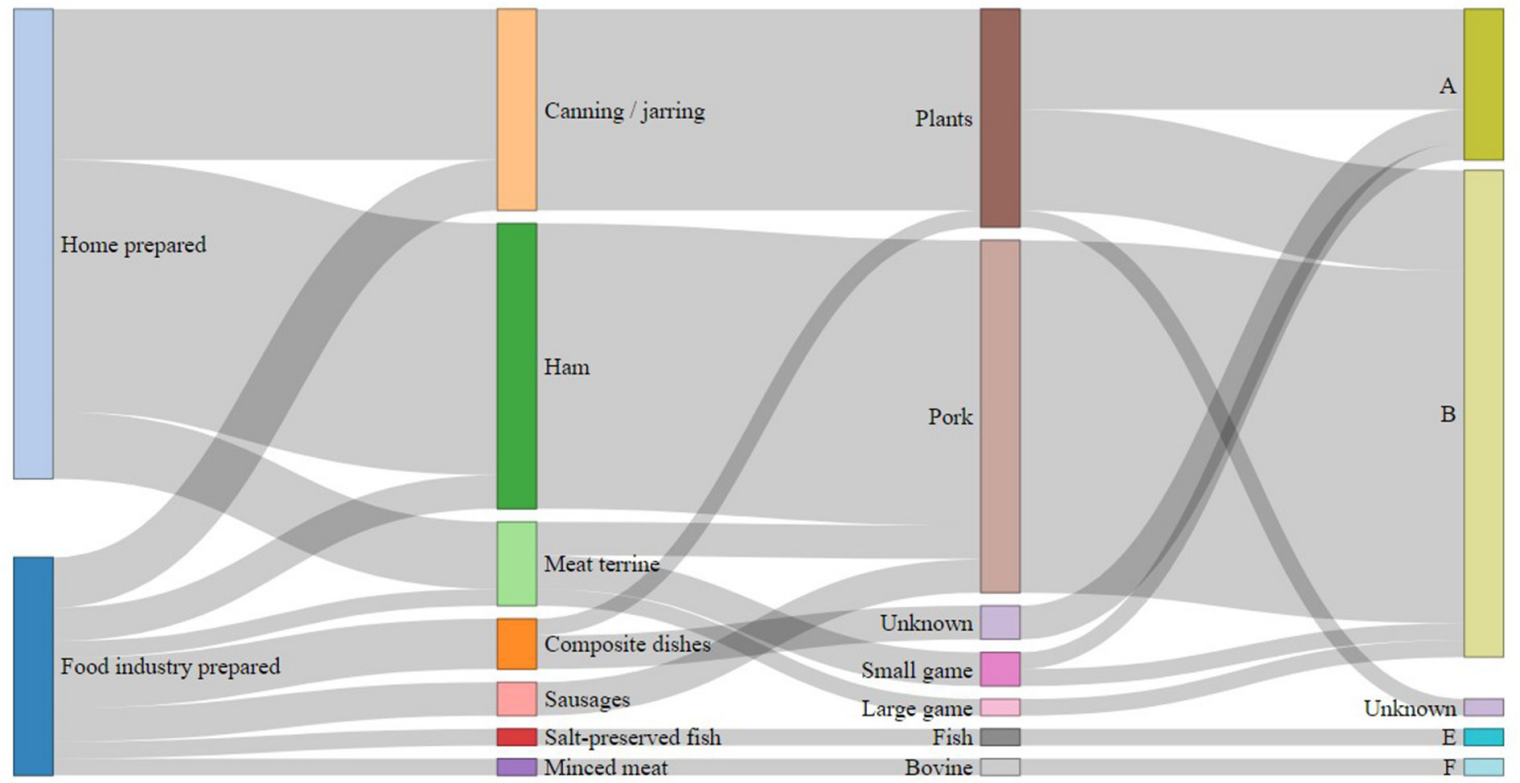
Distribution of foodborne botulism outbreaks with identification of the food source (*n* = 41) in France between 2008 and 2018 according to the type of preparation, nature of the food, origin of the food and type of botulinum toxin.

Due to the unavailability of food for analysis, identification of contaminated food was only possible in 41 (50%) outbreaks (Figure 5). Considering that cases of infectious botulism (intestinal colonization) are rare, those outbreaks are considered to be foodborne even if the food at the origin to BoNT has not been identified. The most common types of food involved in human botulism outbreaks were canned foods and homemade products. The two main food sources were raw ham (*n* = 17) and canned vegetables (*n* = 12). Three composite foods, i.e., smoked fish, salted fish and minced meat, were also the source of botulism outbreaks. For each outbreak with identified food, a detailed description on the foods according to FoodEx2 classification (35), together with the toxin type and the number of cases per outbreak is provided in Supplementary Table 1.

Of the 14 reported cases of infant botulism, 6 were of type A and 8 of type B. All the putative food samples possibly involved and analyzed during the investigations were negative and the origin of these cases remains unexplained.

#### In poultry

**Figure 6:**
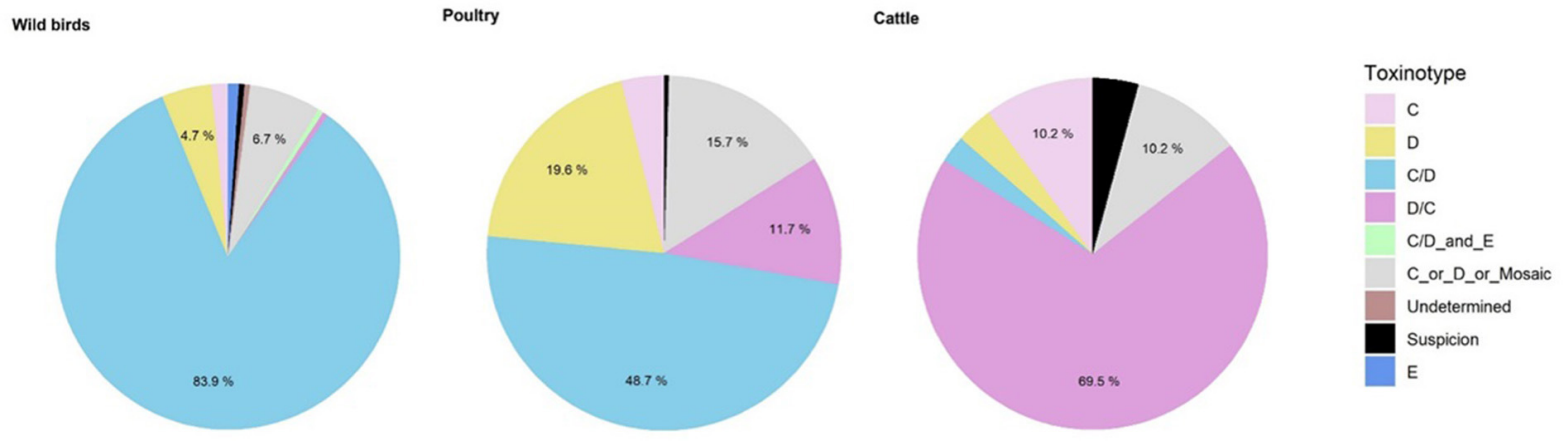
Distribution of botulism toxins from 2010 to 2019 for wild birds (*n* = 193), poultry (*n* = 231) and cattle (*n* = 118).

From 2009 to 2019, the most common BoNT type in poultry was BoNT C/D (*n* = 112, 48.7%), like in wild birds. BoNT D (*n* = 45, 19.6%) and D/C (*n* = 27, 11.7%) were also frequently detected (Figure 6). No BoNT E was recorded in France during the study period.

Based on the data available from the NRC and the NRL, the species most affected by botulism were turkeys (*n* = 41 outbreaks, 51%), followed by birds of the genus *Gallus* (laying hens and broilers) (*n* = 28 outbreaks, 35%). BoNT D/C was more frequently encountered in turkeys than in other species. Among the 49 occurrences with known toxin types, BoNT D/C represented 31% of the outbreaks (*n* = 15). In *Gallus* the majority of the 33 occurrences were due to BoNT C/D (*n* = 28, 85%). For guinea fowl, BoNT C/D was the most common (*n* = 7, 50%). Only three occurrences were observed over the period in ducks, two of which were associated with BoNT C/D.

Of the 64 outbreaks for which information is available, the majority of cases occurred at the end of the breeding period, regardless of the species. The median age of onset of the disease in turkeys (*n* = 37), broilers (*n* = 19) and guinea fowl (*n* = 6) was 88, 43 and 47 days, respectively. Few data are available on the poultry production stages. Of the few data available (*n* = 44), BoNT C, D and C/D were observed in the meat stage for all species. Only one C/D outbreak was observed on a breeding farm. Of the 14 outbreaks that occurred between 2013 and 2019 for which this information is available, half involved certification label or organic poultry, the other seven involved standard or certified poultry.

Most of the outbreaks occurred in Brittany (*n* = 32, 42%). Of the 91 outbreaks studied since 2013, almost half were observed in the third quarter of the year (*n* = 43, 47.3%), with a large number observed in the fourth quarter (*n* = 24, 26.4%).

#### In wild birds

Since the development of laboratory techniques to distinguish mosaic forms and their implementation for routine analysis (2010), botulism type C/D has been the most common (*n* = 162, 83.9%) (Figure 6). Three outbreaks involving *C. botulinum* BoNT E were detected in 2018 in wild birds [mute swan (*Cygnus olor*), mallard duck (*Anas platyrhynchos*) and stork (*Ciconia ciconia*)], always associated with *C. botulinum* C/D.

The bird species most affected by botulism are those belonging to the family *Anatidae* (geese, swans, ducks, etc.) (*n* = 71 outbreaks, 87%). Among the 74 occurrences of toxin types found in *Anatidae*, BoNT C/D was the most frequent (*n* = 71, 96%). Botulism outbreaks in other species of wild birds were less common (*n* = 11, 13%) (*Laridae*: seagulls, gulls, etc. and *Rallidae*: rails, coots, etc.). BoNT C/D was again the most common toxin type in these species.

The cases were distributed across the whole country and were more frequently observed during the third quarter of the year (i.e., July, August, or September) (*n* = 52, 80%). A smaller proportion of outbreaks was observed in the fourth quarter (*n* = 13, 20%). Only one case was recorded in the first quarter of the year.

#### In cattle

For cattle, BoNT D/C was the most prevalent toxin type (*n* = 82, 69.5%) followed by BoNT C (*n* = 12, 10.2%) (Figure 6). No BoNT D outbreaks have been confirmed on cattle farms in France in recent years. Regarding the seasonality of bovine botulism outbreaks, the 36 outbreaks observed appear to be spread over the first (*n* = 8, 22%), second (*n* = 14, 39%) and third quarters (*n* = 9, 25%). A few outbreaks were also observed in the fourth quarter (*n* = 5, 14%), with no evidence of a seasonal effect.

Most outbreaks occurred in Brittany (*n* = 20, 56%). The median age of onset of the disease was 27 months in affected cattle.

## Discussion

The analysis of surveillance data made it possible to assess human and animal botulism in France. Our study confirms that the disease is present in many species, being rare in humans with an occurence of 10 persons affected per year, and much more common in animal species, essentially in birds (wild and poultry) and cattle, which are the two most affected categories of animals. Each year, on average, 10 outbreaks are recorded in the bovine sector, 30 in poultry sector and 20 cases in wild birds, each of which can affect several thousand birds (30).

At the European level, human data come from systems equivalent to the French mandatory reporting system (36, 37). These surveillance systems can be considered as exhaustive for the detection of severe forms of botulism. The number of confirmed cases over the 2011–2018 period was relatively stable with ~100 cases reported per year. The incidence rate in Europe is around 0.02 cases per 100,000 inhabitants, similar to the incidence rate in France (36). The countries with the highest number of confirmed cases are Italy, the United Kingdom, Poland, Romania and France. In Italy, 466 cases of botulism were identified from 1986 to 2015: 93% were foodborne botulism, infant botulism accounted for only 6% of cases and wound botulism for 1% (38). In Turkey between 1983 and 2017, 95 cases of botulism were identified, and the food category primarily responsible for the cases was home-canned vegetables (39). In Ukraine, between 1955 and 2018, 8614 cases of botulism were reported (40).

Infant botulism is the most common form of botulism in the United States and has accounted for 80% of reported cases of childhood botulism worldwide since this form of the disease was first recognized in 1976 (41). It has an average annual incidence rate of 2.1 cases per 100,000 live births (42). A recent review covering the 1976–2016 period identified 1345 cases (6.5 cases/100,000 live births/year) caused by types A, B, Ba, Bf and F in the state of California (43). The average annual incidence rate was calculated at 4.3 cases per million live births in Canada during the period 1979–2019 (44).

For animals, few data of surveillance are available at the global level and most of them come from France, where this disease is particularly monitored in animals. Botulism has previously been reported in 264 bird species representing 39 families (30). *Anatidae* is one of the most affected families, at least in France, as highlighted in our study and in at least one other study (16). In poultry, the species affected by botulism outbreaks are broilers, turkeys, pheasants and, to a lesser extent, ducks, laying hens (raised on litter or free-range only), geese, quails and guinea fowl (2, 16, 45–47). For cattle, only case reports are available in the literature and prevalence has not been reported.

Regarding other animal species, few are affected by botulism in France. A few cases were observed in domestic carnivores (cats and dogs) and only one case was reported in fish during this period. Information on the presence in fish is of great importance, because fish may be naturally affected by type E botulism responsible for human botulism. Mortalities due to botulism type E have been described around the world in wild species (e.g., the round goby *Neogobius melanostomas* in the Great Lakes region of North America, the catfish *Ictalurus punctatus* in the Mississippi Delta in the United States) (48–50). Regularly described on aquaculture farms from the 1960s to the 2000s (especially on trout or salmon farms), botulism outbreaks in aquaculture seem to have become rare, due in part to changes in farming and health management practices. The only relatively recent references, apart from those relating to cases of botulism E affecting fish in the Great Lakes region (48), involve botulism in catfish reported from some farms in the southern United States of North America (51, 52).

Our analysis of occurrences during the period studied here shows that the incidence of human botulism has been relatively stable over time. Similarly, animal botulism also appears to experience relative stability, although there are annual variations for which the origin cannot always be identified. Comparison over a longer period is made difficult by the changes in the animal botulism surveillance system in France over time and especially the significant development of diagnostic methods. Before 2010, the BoNT detection method did not allow the identification of mosaic forms, and different analytical methods were used to differentiate between BoNT types. The characteristics of the tests have also improved, with the optimization of sampling methods (choice of matrices, sampling protocol, transport and storage methods) (2, 46) probably leading to better sensitivity in regards to detection or diagnostic confirmation. Nevertheless, there are still situations, particularly in the bovine sector, where clinical suspicions strongly suggestive of the disease cannot be confirmed by laboratory analyses. For example, sera collected on symptomatic animals are often negative for BoNT using the mouse bioassay (45), probably because BoNT is not circulating any more when the sample is collected. A difference in sensitivity to BoNTs between mice and cattle could also be hypothesized. It has indeed been suggested that cattle are 12.88 times more sensitive to BoNT C than a mouse on a per kilogram weight basis (53), BoNT C has moreover been shown to be the least toxic BoNT types for mice (54). On the contrary, mice are extremely sensitive to BoNT D/C, which harbored the highest toxic activity among tested BoNT types (54). While a difference in sensitivity between mice and cattle may explain the failure of the mouse bioassay to detect BoNT C in serum samples from cattle, this seems unlikely as far as BoNT D/C concerns considering the high sensitivity of mice to this BoNT type. Detection of BoNT-producing clostridia could be sometimes tricky when contamination is low and not homogenous within the matrix. Several matrices (liver, ruminal content, fecal samples…) collected from different symptomatic animals should be analyzed to make sure *C. botulinum* will be detected.

The cases presented in this report correspond to those identified by the NRC and the NRL. In France, any suspicion that is submitted for laboratory diagnosis currently goes through a reference laboratory. The severe forms of human botulism are probably reported exhaustively (mild forms may not be detected in humans, e.g., solely involving digestive discomfort), but it is likely that a certain number of animal botulism suspicions are not reported, and their extent cannot be assessed. This under-reporting is probably limited in the cattle sector. In the poultry sector, because botulism outbreaks occur at the end of the rearing period, we cannot exclude the possibility of flocks being sent to slaughter at the start of an outbreak of botulism. Surveillance of botulism in wild birds, which is based on event-based surveillance, leads to an obvious under-representation of cases, but it is not possible to assess to what extent.

Analysis of the toxin types occurring in France confirmed the predominance of types A and B in human botulism—in both foodborne and infantile cases—and exceptionally type F (55, 56). At the international level, BoNT types that cause human cases are types A and B, followed by E and, occasionally, F. A meta-analysis of outbreaks including 197 outbreaks of foodborne botulism (nearly half of which involved outbreaks in the US) identified BoNT A, B, E, and F as the causative BoNT in 34, 16, 17, and 1% of outbreaks, respectively (57). BoNT B is the most prevalent BoNT in France, like in Poland where type B represented 83% of the cases in 2016 (58). In Italy, from 1986 to 2015, BoNT B was involved in 79.1% of cases (261/330), followed by BoNT A (9.7%, 32/330), with BoNT F, Ab, and Bf, accounting for 0.3 (1/330), 1.5 (5/330), and 0.6% (2/330) of all cases, respectively (38). In Ukraine, BoNT B (59.64%), E (25.47%), and A (7.97%) are the most common, with cases related to BoNT C being very minor (0.56%) and only suspected (40). In North America, foodborne botulism outbreaks originate from vegetables (home-canned), but mostly BoNT E, originating from fish or marine mammals prepared in indigenous communities using traditional methods (e.g., fish fermentation) (42). Similarly, in various Asian countries, outbreaks typically arise from traditional food preparations (59–61).

The C/D mosaic form is the predominant BoNT in birds in France. BoNT C and D are also observed, but to a lesser extent. Other European countries report similar findings on field collections of strains from animal botulism outbreaks (3, 62). Although the majority of bird species are experimentally sensitive to various BoNTs, the only BoNTs naturally involved in outbreaks in birds are BoNTs C, D or their mosaics C/D and D/C, BoNT E and, much more rarely, BoNT A (63). BoNTs C, D or their mosaics C/D and D/C are the most frequent, both in wild and domestic species. BoNT E is less frequently detected, and regularly causes sporadic cases or epizootics in wild fish-eating birds in northern regions, but is rarely the cause of epizootics in farmed species. Type A botulism has only been described a few times in the United States in avifauna including deaths of seagulls in the Klamath River basin in California (63) and it seems to be excessively rare on farms [one outbreak on a broiler farm in the United States, see Graham and Schwarze (64)].

In France, only BoNTs D/C and C have been identified in recent years in bovine botulism outbreaks. In Europe, BoNT D/C is the currently cause of the majority of bovine cases (62, 65). Very rare cases with BoNT A were reported in the middle of the 20th century in France Prévot et al. (66, 67) cited by the French Agency for Food safety and Animal health (68), in zebus (*Bos indicus*) in Brazil (69), in dairy cows in Egypt in 1976 (4) and very recently in the state of New-York in the United States (70). Type B outbreaks have also been described in the literature in dairy herds: in the United States in 1984, 1992 and 2001, in Israel in 2000 (71–74) and in the Netherlands in about 30 dairy herds in 1976 and 1977 in the Netherlands in connection with the incorporation of contaminated brewers' grains in the feed ration (75).

The detailed analysis conducted on NRL data on avian botulism provided interesting details, particularly for poultry farms (species involved, age of onset of cases, dominant toxin types by species, etc.). In our data, there were no differences between males and females in poultry farming. In the literature, males appear to be more affected than females, particularly in turkeys (47, 76, 77). No explanation for this observation has been provided to date. For example, males have a longer rearing period than females, but the impact of this factor on the occurrence of an outbreak of botulism has not been evaluated. Botulism can also occur as a result of stress or a biosecurity failure at the time of removal of the females. Most of the outbreaks in both cattle and poultry are located in Brittany, in an area with a high density of poultry and dairy farms, and a high number of mixed farming, which may explain frequent cross-contamination and this higher prevalence. It is unlikely that there is detection bias, because the level of disease surveillance is the same throughout the country.

It was not possible to conduct a detailed analysis on cattle due to the lack of previous data, available only since 2017. In any case, this analysis remains difficult to conduct retrospectively. The information available is based almost exclusively on more or less complete information forms accompanying the samples sent to the NRL. A standard information form listing the essential data to be transmitted with the samples would facilitate the monitoring of animal botulism in France.

Addressing the study of pathogens not sector by sector but from a global perspective is the basis of the One Health concept. This approach address a health threat at the human-animal-environment interface based on collaboration, communication, and coordination across all relevant sectors and disciplines with the ultimate goal of achieving optimal health outcomes for both people and animals (78). Botulism is part of the European list B of Annex I of the zoonoses Directive (79), surveillance and study of botulism, BoNTs, and BoNT-producing clostridia logically fall under the One Health concept. If botulism is notifiable for humans in Europe, this is not systematically the case for animals. In France, botulism is a notifiable disease, both in humans and animals, regardless of the species affected, which allows for an overall view. The occurrence of botulism cases and outbreaks is closely monitored through a case-based, passive surveillance system. This is a first step in the application of the One Health approach to disease surveillance by juxtaposing animal and human surveillance. In the majority of cases, surveillance systems continue to be developed and operated within a highly sectoral approach (80). But to be effective, the management of complex health issues should shift from isolated, sectoral and linear, to systemic and transdisciplinary approaches to health (81). Our study has shown that human botulism is mostly due to ham (pig sector) and canned vegetables, indicating the importance of collection of surveillance data from food industry, animal sectors as well as surveillance of this pathogen in the environment. These results show that even if surveillance is implemented for both human and animal health, progress are still needed to improve data collection and surveillance of food, feed sectors and environmental contamination.

With the exception of BoNT E, which was exceptionally detected throughout the study period in wild birds, the types of botulism found in animal outbreaks are different from those identified in human outbreaks over the last 10 years in France and no human botulism outbreaks investigated by SPF and the NRC have been linked to animal botulism. But both human and animals are known to be sensitive to some similar BoNT types. As a result, detecting a BoNT E outbreak in wild birds or in poultry, or a BoNT B outbreak in cattle is crucial to prevent any contamination to humans. Furthermore, there are currently very few cases of type C, D, C/D, D/C in humans. It is important to continue to monitor over time that this is still the case. Early detection of zoonotic pathogens through enhanced laboratory capacity and surveillance at the animal–human interface is a crucial step toward controlling and preventing zoonoses (82).

Given that botulism is ubiquitous in the environment and can cause disease in both humans and animals, it is essential to enhance links between human and animal surveillance systems. Accordingly, in line with the One Health concept, this study presents the first integrative approach to the routine surveillance of botulism in humans and animals in France.

## Data availability statement

The original contributions presented in the study are included in the article/Supplementary material, further inquiries can be directed to the corresponding author.

## Author contributions

SL, CLM, RS, LG, and CM were responsible for the concept and design of the study, interpretation of results, writing, and critical review of the manuscript. CLM, RS, and LG were responsible for data collection. CL and LG were responsible for statistical analysis. CLM, KP, PK, and FM were responsible for reviewing and editing the manuscript. All authors contributed to the article and approved the submitted version.

## Funding

This work was supported by the French Ministry of Agriculture and Food.

## Conflict of interest

The authors declare that the research was conducted in the absence of any commercial or financial relationships that could be construed as a potential conflict of interest.

## Publisher's note

All claims expressed in this article are solely those of the authors and do not necessarily represent those of their affiliated organizations, or those of the publisher, the editors and the reviewers. Any product that may be evaluated in this article, or claim that may be made by its manufacturer, is not guaranteed or endorsed by the publisher.

## Supplementary material

Supplementary material 1 Foods involved in human botulism outbreaks from 2008 to 2018.
